# Students’ perceptions of the effectiveness of additional language tuition in the University of Cape Town MBChB programme: A descriptive cross-sectional study

**DOI:** 10.4102/phcfm.v11i1.2121

**Published:** 2019-10-17

**Authors:** Zahraa Mohamed, Stephanie Roche, Joel Claassen, Zukile Jama

**Affiliations:** 1 Faculty of Health Sciences, University of Cape Town, Cape Town, South Africa; 2 Afrikaans and Netherlandic Section, School of Languages and Literatures, Faculty of Humanities, University of Cape Town, Cape Town, South Africa; 3 Faculty of Humanities, University of Cape Town, Cape Town, South Africa

**Keywords:** student perceptions, communications skills, languages barriers, additional languages, doctor-patient communication, Xhosa, Afrikaans

## Abstract

**Background:**

Language barriers between doctors and patients have been shown globally to negatively affect the quality of health care and infringe on basic patient rights. In response to these challenges, the Division of Family Medicine at the University of Cape Town (UCT) integrated career-oriented Afrikaans and Xhosa communication skills classes into the MBChB degree programme in 2003.

**Aim:**

To measure students’ perceptions of the effectiveness of the language communication skills classes in creating multilingual medical practitioners in the South African context and compare these perceptions between the Afrikaans and Xhosa courses.

**Setting:**

The study was conducted on the Health Sciences campus of the University of Cape Town, South Africa.

**Methods:**

The study was a cross-sectional survey. During March 2017, access to an online structured questionnaire was provided to 600 randomly selected medical students from second to sixth year at the UCT.

**Results:**

The response rate was 43.7%, and students reported a much higher baseline level of Afrikaans compared to Xhosa (99.0% vs. 42.7%). Respondents reported a lack of confidence in the clinical sphere for both languages (Afrikaans 51.5%; Xhosa 60.0%) and a lack of communicative ability (Afrikaans 35.3%; Xhosa 67.2%) as major barriers to patient communication.

**Conclusions:**

Respondents overwhelmingly agreed that second language learning is valuable for their future as medical practitioners, but did not feel that they are developing sufficient communicative competence. The courses need to be re-evaluated to account for the lower level of pre-MBChB Xhosa exposure, as compared to Afrikaans. Increased time allocated to languages, increased attention to cultural issues and informal variants, and redesigning assessments to better reflect students’ abilities are all potential recommendations.

## Introduction

Language barriers between doctors and patients pose a significant health challenge globally, in Africa and in South Africa. It has been shown that these barriers decrease the quality of care and infringe on the basic patient rights.^1,2,3,4^ Language barriers are associated with decreased access to health care, unnecessary testing and increased hospital stays, highlighting both social and economic effects.^5^ Improved patient communication also leads to improved patient satisfaction and thus adherence to treatment, which improves health outcomes on an individual and societal level.^6^ Informed consent is inevitably affected, with patients agreeing to procedures without fully understanding the intricacies involved.^4,7,8,9^

There is extensive literature from around the world describing the existence of language barriers in health settings, which is largely descriptive, but there is only limited information available on the interventions that might be used to overcome these barriers.^10^ Interestingly, more research is becoming available on health care professionals’ perceptions of language barriers, which our research will contribute towards, but there is still only limited evidence on the feasibility and effectiveness of interventions.^10^ Various studies describe the limitations of using interpreters, including ethical issues around confidentiality; however, there is a lack of research investigating language tuition for medical students as another tool to overcome language barriers in clinical contexts.^4,7,11^ Although our research is not an interventional study, we aim to provide a better understanding of the current student perceptions in order to provide a basis for future research, which can then assess the effectiveness of small group language teaching in medical schools as an intervention to reduce language barriers in the health care setting.

As in other African countries, the challenge to the South African health system is significant because of the language barrier, as there are 11 official languages. There are over 8 million home-language Xhosa speakers and almost 7 million home-language Afrikaans speakers.^12^ Despite this, the majority of South African health professionals, excluding practicing nurses, are unable to communicate in any of the indigenous languages.^13^ In a study at the Red Cross War Memorial Children’s Hospital, it was found that most interviews were not conducted in the patient’s home language and occurred without the use of an interpreter.^8^ Patients reported that language, along with cultural factors, formed an immense barrier to health care with more than 70% being dissatisfied with communication during interviews.^7,13,14,15^

The responsibility of ensuring successful communication is placed on patients, who are expected to understand English.^7,16^ This expectation poses a threat to both cultural diversity and the dismantling of Western cultural norms.^7^ In South Africa, the negative relationship between previous disadvantage and English proficiency means that language barriers in the clinical setting further marginalise illiterate and uneducated South Africans.^7^ As discussed by Meuter et al., language barriers in a patient–doctor relationship represent the power dynamic and can be used to enforce or manipulate the power dynamic; the importance of recognising and addressing this is essential when taking into account the already deeply entrenched inequality in South Africa.^8^ Despite a constitutional and legislative framework, there has been slow progress in developing multilingualism on a broad scale, a process that must occur in order to produce medical doctors that are communicatively competent and patient-centred.^17,18,19^

In 2003, the Faculty of Health Sciences at the University of Cape Town (UCT) started towards this goal by introducing career-oriented Afrikaans and Xhosa communication courses to MBChB students from year 1 to year 4. In year 1, students who self-identify as beginners in either Xhosa or Afrikaans (i.e. Common European Framework pre-level A1, Interagency Language Roundtable (ILR) scale Level 0, American Council on the Teaching of Foreign Languages (ACTFL) Novice level, or Defense Language Proficiency Test (DLPT) Level 0) receive a 12-week introductory course. During years 2 and 3, all medical students attend weekly, face-to-face, small group language tutorials and undergo assessments through Objective Structured Clinical Evaluations (OSCEs) in the Family Medicine course, *Becoming a Doctor*. In year 4, the module *General Medicine* includes a language component that is tested in a clinical setting. There has never been an independent evaluation of student perceptions regarding these courses.

This study aimed to investigate medical students’ perceptions of their additional language learning within the UCT MBChB programme and their subsequent communicative competence. In addition, and in line with recent student-led demands to move towards more Afrocentric curricula, the study aims to compare the results for Afrikaans and Xhosa to evaluate whether the current programme is producing graduates with competencies in both Afrikaans, a language historically used in higher education, and Xhosa, an indigenous language historically marginalised in South African universities.^20,21,22,23^

## Methods

### Study design

A descriptive, cross-sectional study design was selected, using a questionnaire consisting of multiple-choice questions.

### Setting

The study was undertaken at the UCT, Observatory, Cape Town, South Africa.

### Study population

The study population comprised students registered for the MBChB programme at the UCT.

#### Inclusion criteria

The inclusion criteria were participants enrolled in the MBChB programme at UCT for the 2017 academic year, completing one of years 2–6.

#### Exclusion criteria

None.

### Sample size and sampling

A random sample of 600 was chosen from a total population of approximately 1200 medical students, and the sample was stratified by year of study, with 120 chosen from each year’s cohort between years 2 and 6. This allowed for a 5% level of precision and 95% confidence interval. Students were randomised using Excel-generated numbers.

### Data collection

Data were collected through the use of an online questionnaire. This questionnaire was developed by the authors, in conjunction with experts in the fields of health care and language education from the UCT, as no existing validated questionnaire was available to assess the unique teaching and learning programme at this university.

The first six questions were used to ascertain students’ prior exposure to Afrikaans and Xhosa, by assessing both the self-reported level of exposure and competency as well as the type(s) of exposure, for example, social environments versus formal teaching. This was followed by questions assessing how students perceive specific aspects of the language courses, ranging from class size to time given to cultural aspects of the language. Students were then asked to assess the real-life value of their language teaching and to provide an estimate of how often and to what extent they communicate in their patients’ home languages. Finally, students were asked to identify barrier(s) that prevent them from communicating in a patient’s home language, with an ‘other’ option to record additional barriers they had experienced.

The questionnaire was made available on Google Forms and was emailed to students who had been randomly selected to participate from a database of student numbers. Posters were put up around the Health Sciences Faculty, encouraging all students to check their emails to determine if they were selected. Students were given 1 month (March 2017) to complete the questionnaire, and three reminder emails were sent 9, 23 and 25 days after the initial email.

### Data analysis

Data from Google Forms were automatically captured in Google Sheets, after which they were transferred to Microsoft Excel 2016 for analysis. For responses to questions with the options ‘strongly agree’, ‘agree’, ‘neutral’, ‘disagree’ and ‘strongly disagree’, the ‘strongly agree’ and ‘agree’ categories were grouped together for presentation in table and chart form. Microsoft Excel 2016 was used to calculate the frequency of the different responses and to present the data in chart form.

### Ethical consideration

Ethics approval was obtained from the Human Research Ethics Committee at the University of Cape Town (HREC REF: 155/2016). No incentives were provided to complete the questionnaire apart from the possibility of improvements in the current language programme. Students were required to consent to participate in the study, and both the consent forms and questionnaires were offered in English, Afrikaans and Xhosa (Appendix 1).

### Results

The overall response rate was 43.7%. The sample comprised a fairly even spread of students across the years, with the lowest number of responses from final year students (11.8%). The majority of students (95.8%) reported being fluent in English. Over a third regarded themselves as fluent in Afrikaans, with 5.73% of all respondents reporting Afrikaans as their home language. Regarding Xhosa, only 11.5% of students considered themselves to be fluent, despite having the same proportion of home language speakers (5.73%). Zulu (15.7%) and Sotho (10.7%) were the only other languages that amounted to greater than 10.0%.

While 99.0% of the respondents reported having had exposure to Afrikaans prior to the MBChB programme, only 42.7% of all respondents reported any prior exposure to Xhosa (Table 2). Over 85.0% of respondents completed Afrikaans as a school subject, while less than a third reported having done Xhosa at school. There were similar levels of extra-curricular and university-level course exposures between Xhosa and Afrikaans, but significantly less exposure to spoken Xhosa (12.2% vs. 20.6%).

Participants were asked to grade various factors within the language programme as adequate or inadequate for facilitating their language learning. Just over a third agreed that there is sufficient focus on non-standard varieties of Afrikaans, with less than a quarter reporting sufficient attention given to cultural issues. Regarding the Xhosa classes, only a quarter reported sufficient focus on non-standard varieties, with slightly more students (34%) agreeing that there is sufficient attention provided to cultural aspects.

Slightly less than half of the participants felt that the assessments are appropriate to assess their Afrikaans communicative abilities, whereas less than a quarter of students felt that Xhosa assessments were valid, despite both assessments being offered in the same format (OSCEs). Although half of the respondents found Afrikaans tutorials useful for real-life clinical settings, only 38.6% of students felt the same about Xhosa tutorials.

**TABLE 1:** Sample population characteristics.

Category	*n*	%
**Year of study (*N* = 262)**		
Second year	56	21.4
Third year	59	22.5
Fourth year	68	26.0
Fifth year	48	18.3
Sixth year	31	11.8
**Language fluency (*N* = 262)**		
English	251	95.8
Afrikaans	95	36.3
Xhosa	30	11.5
Zulu	41	15.7
Sotho	28	10.7
Tswana	17	6.5
Tsonga	6	2.3
Venda	6	2.3
Sepedi	2	0.76
Ndebele	1	0.4
Swati	0	0.0
Other†	20	7.6

† , Bangla, Chewa, Chinese, Dutch, French, German, Gujarati, Hindi, Korean, Spanish, Swazi and Yao.

**TABLE 2:** Level of exposure to Afrikaans or Xhosa prior to MBChB, *n* = 262.

Exposure	Afrikaans		Xhosa	
	*n*	%	*n*	%
None	26	1.0	150	57.3
Primary or high school subject	225	85.9	73	27.9
Extra-curricular course	4	1.5	4	1.5
University-level course	4	1.5	14	5.3
Wide exposure to spoken Afrikaans or Xhosa	54	20.6	32	12.2

Figure 1 shows that although there were nearly equal numbers of Afrikaans and Xhosa home language speakers (5.7%), over 57.0% of the students were first- or second-language Afrikaans speakers, whereas less than 10% of students were first- or second-language Xhosa speakers.

**FIGURE 1:**
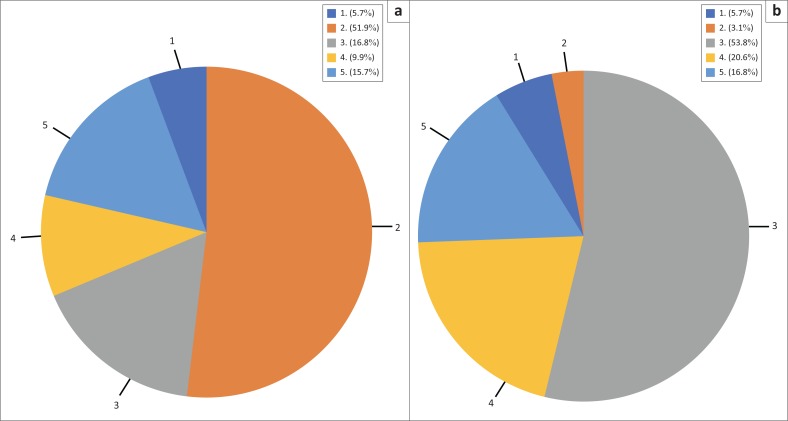
(a) Proportion of home language versus (b) additional language speakers, comparing Afrikaans and Xhosa.

### Proportion of interactions in which students speak Afrikaans or Xhosa with home language Afrikaans or Xhosa patients

The students reported that only 27.7% of them converse with the majority of Afrikaans patients in Afrikaans, and only 20.8% of them converse with the majority of Xhosa patients in Xhosa. Over 50% of students reported speaking to few or no Afrikaans patients in Afrikaans, similar to the responses regarding Xhosa-speaking patients (58.5%).

### Barriers to patient communication

**FIGURE 2:**
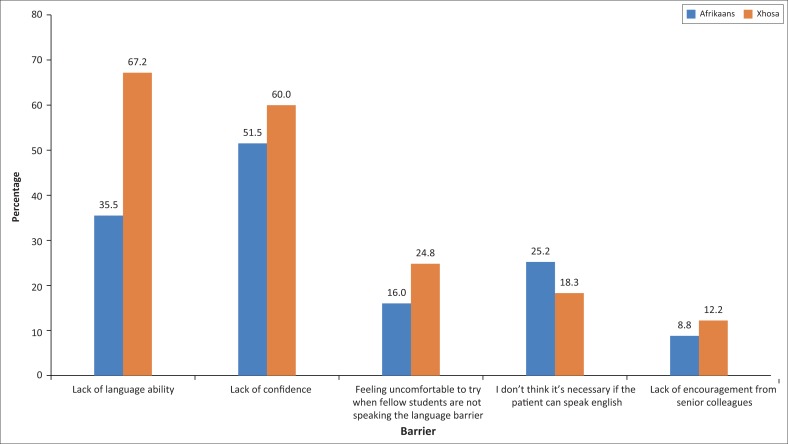
Barriers preventing students from speaking Afrikaans or Xhosa to home language patients, *n* = 262.

Figure 2 shows that the most frequently identified barriers to speaking Afrikaans were a lack of confidence (51.5%) and a lack of language ability (35.5%). A much larger proportion of students reported the lack of language ability as a barrier to speaking Xhosa (67.2%), which was followed by the lack of confidence (60.0%). All other barriers were experienced by less than 30% of students.

**FIGURE 3:**
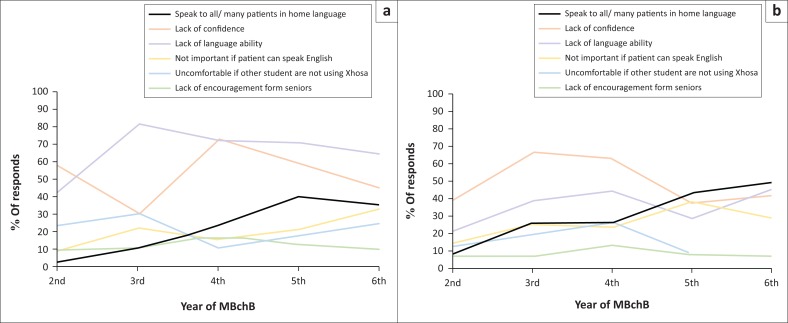
Year-by-year analysis of the proportion of patients spoken to in their home languages: (a) Xhosa and (b) Afrikaans, as well as the prevalence of the reported barriers.

Figure 3 provides a more longitudinal perspective across the current years of study, showing the proportion of students in each year, who reported speaking to patients in the patients’ home languages, as well as how respondents view the barriers to using Xhosa and Afrikaans. There is an increasing trend in the proportion of patients spoken to in their home language for both Xhosa and Afrikaans, although there is a slight decrease in the sixth year for Xhosa. In terms of the barriers for Xhosa, the reported lack of confidence and lack of language ability decreased between the fourth and the sixth year. There was a lower purported lack of confidence in the third year, but otherwise the trend was similar to the lack of language ability. For Afrikaans, the lack of confidence and lack of language ability similarly decreased between the third/fourth and the sixth year. For both Afrikaans and Xhosa, it seems that the lack of importance if patients can speak English increased with advancing years, and the lack of encouragement from seniors remained relatively stable. Discomfort using Xhosa when fellow students were not was highest in the early years for Xhosa, decreased in the fourth year, and increased thereafter. The opposite was true for Afrikaans.

Table 4 shows that more than 75% of students are comfortable greeting Afrikaans and Xhosa patients in their home languages. With each subsequent area of patient interaction, an incrementally lower proportion of students reported feeling comfortable speaking to patients, for both languages. The greatest disparity between Xhosa and Afrikaans was in understanding responses, with 61.8% of students reporting that they were comfortable in understanding Afrikaans responses, whereas only 26.3% of students were comfortable in understanding Xhosa responses. The area in which the most students struggled was breaking bad news, for both Afrikaans (35.1%) and Xhosa (14.1%).

### Students’ perceptions around the value of language learning

The vast majority of the respondents (85.12%) stated that they believed that learning Xhosa and/or Afrikaans during the MBChB programme is valuable to their future as health care professionals, with 7.25% of the respondents not agreeing that the language courses are valuable, and 7.63% of the respondents were reportedly neutral. Conversely, only 31.0% of the participants reported studying languages on a weekly basis, while around 46% of the respondents admitted to only preparing for assessments.

**TABLE 3:** Current language programme factors that students perceive as facilitating learning (*N* = 262).

Category	Afrikaans		Xhosa	
	*n*	%	*n*	%
Sufficient focus on non-standard varieties†	95	36.3	67	25.6
Sufficient attention to cultural issues	59	22.5	89	34.0
Validity of assessments	119	45.4	66	25.2
Clinical or real-life relevance of tutorials	133	50.8	101	38.6

† , Non-standard varieties refer to informal variants of Xhosa and Afrikaans, including Cape (‘Kaapse’) Afrikaans and ‘kasi’ lingo.

**TABLE 4:** Areas in which students are comfortable speaking, *n* = 262.

Area of interaction	Afrikaans		Xhosa	
	*n*	%	*n*	%
None	4	1.5	9	3.4
Greeting	218	83.2	208	79.4
Taking history	195	74.4	156	59.5
Examination	157	59.9	82	31.3
Understanding responses	162	61.8	69	26.3
Investigations, treatment	107	40.8	43	16.4
Breaking bad news sensitively	92	35.1	37	14.1

## Discussion

### Language exposure prior to the MBChB programme

An important pre-supposition confirmed in this study was the major difference in prior exposure to Xhosa versus Afrikaans (Table 2); 90% of students reported having some exposure to Afrikaans, compared to less than 30% for Xhosa. This low level of exposure could be attributed to historical language imbalances within schools; unlike Afrikaans, Xhosa has not been a compulsory language for learners at primary schools and high schools in the Western Cape. In addition, there was also a significant discrepancy in spoken language; 21% of students had exposure to spoken Afrikaans, but only 12% had exposure to spoken Xhosa.

Although there were equal numbers of Afrikaans and Xhosa home language speakers, almost 60% of students were first- or second-language Afrikaans speakers, whereas less than 10% of students were first- or second-language Xhosa speakers. Because of this differing baseline exposure, more intensive and earlier exposure is recommended for Xhosa for beginners, who comprised the majority of the sample.

Another interesting area of focus could be exposure in the medical environment, particularly after students have graduated. Although this research looks at prior exposure to Xhosa and Afrikaans, largely during school and social environments, and exposure during the MBChB programme, doctors entering the health care system will continue to learn and experience language in the South African health care system. As a way to improve doctors’ cultural competence, Betancourt et al. recommend making changes at a more systemic level, which would include increasing the number of doctors and health care facility leaders who represent traditionally marginalised language and cultural groups.^6^

### Factors that affect student language learning

#### Exposure to non-standard varieties of Afrikaans and Xhosa

Just over a third of respondents agreed that there was sufficient exposure to non-standard Afrikaans. This low percentage could possibly be attributed to the fact that the course is career-oriented, resulting in more teaching of the standard Afrikaans variant. Despite the institutional commitment to make students aware of Cape Afrikaans,^24^ respondents clearly felt that a greater effort should be made to improve students’ confidence in understanding Cape Afrikaans.

For Xhosa, only a quarter of students claim to have adequate exposure to non-standard varieties. This could relate to the low level of prior exposure, meaning that students are still learning the basics and have not been able to focus on the local nuances of spoken Xhosa. This inadequate exposure to non-standard variations may also explain why students have such difficulty communicating with real-life patients and why almost 75% of students report being unable to understand their patients’ responses (Table 4). As with Afrikaans, more effort can be made to provide variations of vocabulary and phrases that students might encounter in local communities.

#### Cultural issues

Being able to understand and engage with cultural components of language is an important part of developing cultural competence, a critical component of language learning.^25,26,27,28,29^ Only 22.5% of respondents felt that cultural issues are appropriately addressed during the Afrikaans communication skills course. Although still a low proportion of students, there were significantly more students who agreed that Xhosa classes provide adequate attention to cultural issues (34%). Importantly, this result does not account for the cultural nuances shared through language teaching in itself, given that language is a primary purveyor of culture^30^ and inherently conveys cultural competency.

Interestingly, the idea that specific cultural information should be ‘taught’ is falling away in health professionals’ education. Traditionally, in an attempt to improve cultural competence, health care professionals were provided with commonly held beliefs of a community. However, it is becoming increasingly clear that this traditional method used to improve health care provision to patients from a range of backgrounds is ineffective, promotes the use of stereotypes and reinforces prejudice. Newer approaches focus on communication as the key aspect of culturally competent medical care – by being able to effectively communicate and understand an individual patient’s belief systems and preferences, patients from a wide range of backgrounds can be equally cared for and respected. Although students may have felt dissatisfied with the limited cultural information provided during classes, incorporating this new approach towards cultural competence in language classes may change students’ perceptions on what it means to be a culturally competent doctor and also encourage language learning.^6^

#### Language assessments

The vast majority of students do not feel that the form of assessment is a valid tool to assess their communicative competence, with significant discrepancy between the languages despite both assessments being offered in the OSCE format. This discrepancy is likely related to the greatly differing baseline and warrants a review of the current assessment standards and marking rubrics. The review could be informed by further research and student discussion groups to better understand why students do not see these assessments as valid and how other assessment techniques could be employed.

#### Clinical utility of language teaching

It is very concerning that a significantly low proportion of students believe that Afrikaans (50.9%) and Xhosa (25.2%) tutorials are relevant for real-life clinical situations, if this is the overarching goal of the programme. It is therefore essential to work with both doctors and medical students to create courses relevant to the clinical setting, which could involve discussion groups as mentioned above.

These results were echoed by the low proportion of students who actually speak to home language Afrikaans and Xhosa patients in their home languages (27.7% and 20.8%, respectively). For both languages, lack of confidence was stated as a major barrier. This likely suggests that the current teaching programme does not sufficiently support the application of students’ learnt communication skills. Future changes could include more frequent role-plays during class, in order to simulate real-life patient encounters. More frequent summative assessments or the addition of regular formative assessments, with specific and goal-directed feedback from tutors or peers, could also encourage confidence while improving students’ communicative competence. More students reported lack of confidence as a barrier to speaking Xhosa than Afrikaans, which suggests that the low level of confidence is not simply related to the subject matter in the clinical setting, which often involves very personal discussions with patients, difficult even in one’s home language. This discrepancy is likely related to the significantly lower level of prior Xhosa exposure, and the interventions mentioned above would be particularly important in the Xhosa curriculum design. Providing immersion learning opportunities for both languages may also be useful in overcoming this problem, and the feasibility of providing this opportunity to students should be explored further.

For Xhosa, lack of language ability was the most commonly identified barrier to communication (67.2%). Looking at the specific areas in which students are not comfortable communicating gives some further insight into the problem and can be used to guide curriculum adjustments for both languages. For Xhosa, the only area in which the majority of respondents felt comfortable was greeting a patient (79.4%). However, all of the components of the interview are essential in caring for patients and require adequate communication skills (particularly for informed consent); the fact that most students do not feel equipped is indicative of the necessity to re-align the programme outcomes to achieve the goals of clinical communication and to provide intensive courses to improve baseline Xhosa communication skills.

For Afrikaans, more than half of the students felt comfortable in greeting (83.2%), taking a history (74.4%), performing an examination (59.9%) and understanding responses (61.8%). Although this is in keeping with the findings of a higher baseline exposure, it also specifically identifies the need to better address the topics of explaining investigations and breaking bad news.

Figure 3 showed a more longitudinal picture over the years of MBChB of the proportion of Xhosa and Afrikaans patients spoken to in their home languages and the reported barriers. As shown in Table 1, the number of respondents from each year was small, with the smallest proportion of responses from the sixth year. As a result, this analysis is somewhat limited. It is not surprising that the proportion of patients spoken to in their home languages increases as the years progress, as students have more experience both in speaking to patients generally and with using the two languages. The decrease in the sixth year for Xhosa could potentially be explained by the rise in the perceived lack of importance of speaking Xhosa, if patients are able to speak English, as well as the rising discomfort in speaking Xhosa when fellow students are not. This finding suggests that extending language tutorials to include years 5 and 6 could lead to an increase in the proportion of Xhosa patients spoken to in their home language. This can be contrasted with Afrikaans where, after the third or the fourth year, the proportion of Afrikaans patients spoken to in Afrikaans increases while the two major barriers, lack of confidence and lack of language ability, decrease. Measures to limit these barriers were mentioned in the previous section to improve.

### Student views on the usefulness of language education

The vast majority of students believe that learning Xhosa and/or Afrikaans during the MBChB programme is valuable. This is an encouraging response, which emphasises the need to address the above-mentioned factors in order to tailor the language courses to student needs, with special focus on improving both students’ confidence and language abilities.

### Study limitations and strengths

The study’s strengths include the large sample size and the use of anonymous online questionnaires, which allowed students to provide more accurate information about their personal experiences without fear of academic or personal consequences, reducing response bias. Both students and staff were involved in the design and implementation of this research, providing important insight from both perspectives.

While this study outlines specific areas in which the current additional language communication skills courses can improve, it does not investigate students’ views around potential solutions, and all recommendations are based on the authors’ interpretations of the student responses. In addition, the nature of a cross-sectional study is that it provides a snapshot view; we were not able to examine differing student perceptions over time. However, comparison of students across the years of study did provide some insight into how student perceptions of their language education, and their own performance, might change as they progress through the MBChB programme. The response rate was quite low at 43.7%, which will have also influenced the results through self-selection bias, despite efforts to reduce this bias through random selection of 120 students per year. Students with a particular interest in language education, or passion for language learning, may have been more likely to respond to the survey had they been selected and may have skewed the results by providing more positive responses regarding their abilities, confidence levels, frequency of patient home language communication and belief in the importance of language learning. Contrastingly, students who felt disappointed by their language education and frustrated by communication difficulties may have also been motivated to respond, resulting in more negative responses. The group of students who responded to the questionnaire was also largely constituted of Xhosa beginners, which may have led to biased results that do not necessarily represent the views of Afrikaans beginners and should be taken into account when working on future curriculum design.

Another limitation of the study is the focus on medical students within the MBChB curriculum; no students from the health and rehabilitation courses were included. Their additional language communication skills courses differ from those provided to medical students, and a comparative study may be useful. Further research could also include comparing the UCT language programme outcomes to those at other South African universities. An interventional study would also be useful to identify better ways of implementing language teaching into the MBChB curriculum. After changes have been made based on the above findings, this study could be repeated to assess students’ perceived outcomes in the clinical setting.

## Conclusion

Language barriers faced by health care professionals in South Africa are extensive and well described in recent literature, and the UCT Division of Family Medicine in the Health Sciences Faculty has taken on the challenge by offering career-oriented communication classes for Afrikaans and Xhosa since 2003. In this cross-sectional study, students have overwhelmingly agreed that learning Xhosa and/or Afrikaans is valuable for their future clinical practice. However, there are major limitations in the way the programme is currently offered.

Firstly, the major difference in exposure to Afrikaans and Xhosa prior to the MBChB programme needs to be carefully considered when implementing and evaluating the courses. Further intensification of the first-year course for Xhosa beginners is recommended to address the discrepancy between language outcomes.

Specific problematic areas identified for both Afrikaans and Xhosa include the limited exposure to non-standard varieties and cultural factors, and the reduced validity of assessments. Increasing the focus on non-standard varieties, immersion-type learning and redesigning assessments are all recommendations that can add value and assist in achieving the course outcomes. The Faculty of Health Sciences has, in 2018, introduced optional, additional weekly tutorials for students in years 1 and 2. Although this tutorial programme has the potential to provide additional support to students and meet the above-mentioned needs, it must be independently evaluated regarding student attendance and outcomes.

Importantly, students do not feel that the language programme is leading to adequate clinical communicative competence. Discussion groups with clinicians and students could be used to better direct the focus of language tutorials and guide curriculum design. The Afrikaans and Xhosa language courses need to be reworked to improve students’ language ability and confidence to better meet the goals of clinical communicative competence.

## Figures and Tables

**FIGURE 1-A1:**
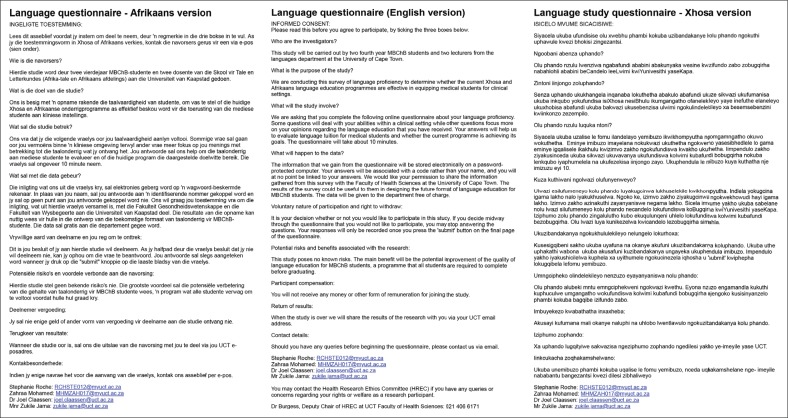
Language study questionnaires.
